# Complements and Their Role in Systemic Disorders

**DOI:** 10.7759/cureus.52991

**Published:** 2024-01-26

**Authors:** Samuel Sherng Young Wang, Haoming Tang, Marcus Wing Choy Loe, See Cheng Yeo, Muhammad M Javaid

**Affiliations:** 1 Nephrology, Tan Tock Seng Hospital, Singapore, SGP; 2 Medicine, Duke-National University of Singapore Medical School, Singapore, SGP; 3 Medicine, Monash University, Melbourne, AUS; 4 Medicine, Deakin University, Warrnambool, AUS; 5 Renal Medicine, Woodlands Health, Singapore, SGP

**Keywords:** therapeutics, diagnosis, pathophysiology, systemic disease, acquired deficiency, congenital deficiency, complement

## Abstract

The complement system is critical to the body's innate defense against exogenous pathogens and clearance of endogenous waste, comprising the classical, alternative, and lectin pathways. Although tightly regulated, various congenital and acquired diseases can perturb the complement system, resulting in specific complement deficiencies. Systemic rheumatic, neurological, ophthalmological, renal, and hematological disorders are some prototypical complement-mediated diseases. An adequate understanding of the mechanisms of the normal complement system and the pathophysiology of complement dysregulation is critical for providing diagnostic clues and appropriately managing these conditions. This review guides clinicians in understanding the role of complement factors in systemic diseases and what diagnostic and therapeutic options are available for complement-mediated disorders.

## Introduction and background

The complements are essential components of the innate immune system, the first-line defense against microbes and damaged host cells. The complement system comprises over 50 proteins that exist as soluble or membrane-bound proteins that are regulators or receptors in an enzymatic cascade. The complement system contributes to immune surveillance and mediates opsonization, a process of tagging foreign pathogens with C3b fragments to facilitate their elimination by phagocytes. The activated complement factors recruit and activate immune cells, leading to subsequent cytotoxic destruction of foreign microbial pathogens and elimination of cellular debris. Complements are also crucial in bridging innate and adaptive immunity by augmenting antibody-mediated responses [1].

Due to the complement system's pro-inflammatory and potentially damaging effects, the system is tightly regulated to avoid damage to the healthy host cells [2]. Both complement deficiencies and overactivation can affect normal immune regulation, resulting in various disorders. Lack of appreciation of the possibility of complement-mediated disorders and poor understanding of the pathophysiological processes involved lead to delayed or misdiagnosis. A better understanding of the complement-related disorders and a high index of suspicion is needed for proper management. This article intends to provide an overview of the normal activation and inactivation of the complement system. It discusses the role of the dysregulated complement system in various inflammatory and systemic diseases.

## Review

An overview of complement activation and inactivation

**Figure 1:**
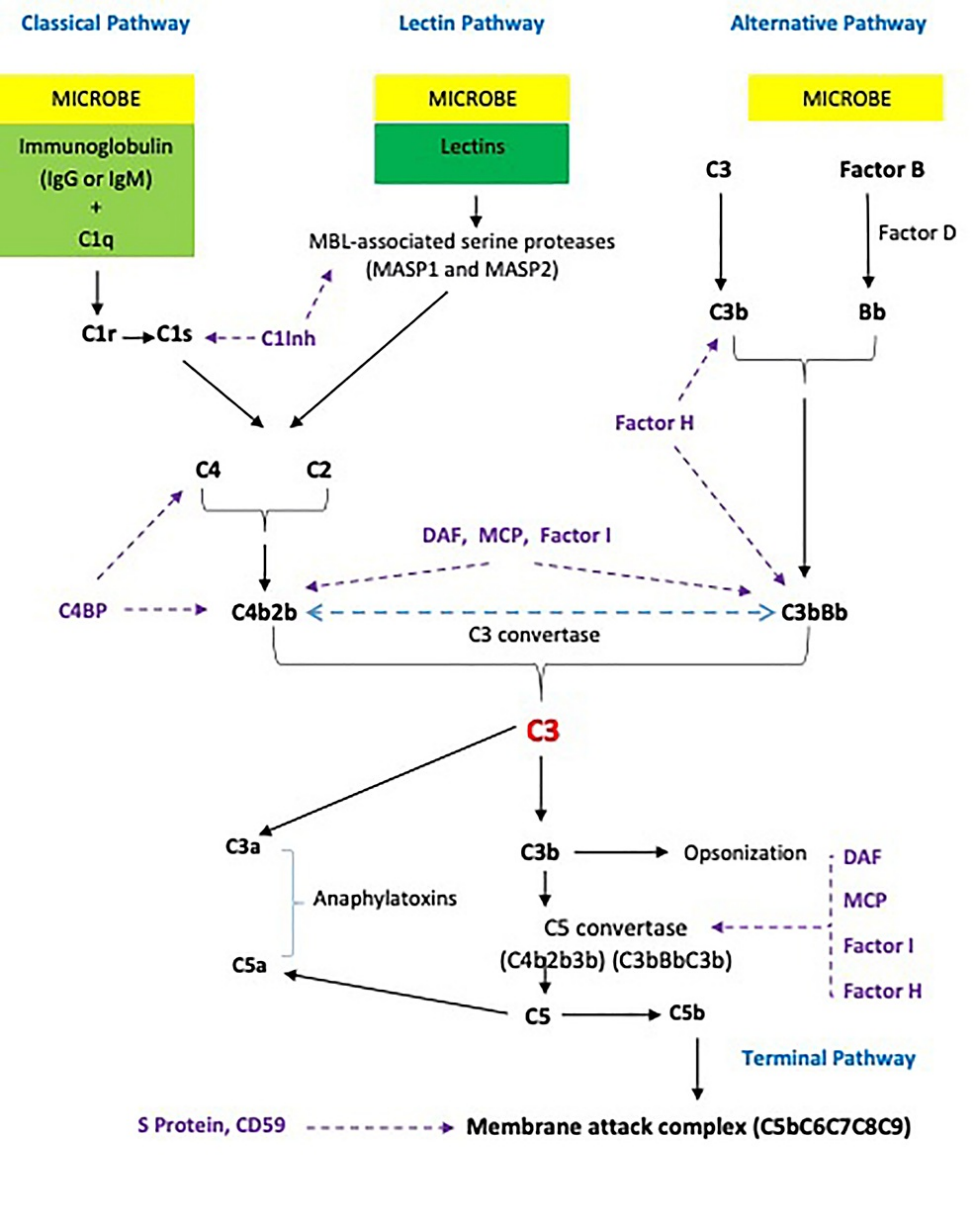
A schematic figure of the complement system regulation Created by Muhammad M Javaid. C1Inh: C1 inhibitor; C4BP: C4b-binding protein; DAF: decay-accelerating factor; MCP: membrane cofactor protein; MBL: mannose-binding lectin; MASP: mannose-binding lectin-associated serine proteases. Regulators (inhibitors and inactivators) are shown in purple.

Three different pathways, classical, alternative, and lectin, activate the complements (Figure 1). Each pathway is activated differently, with all three converging at the C3 step with proteolytic cleavage of C3 to generate C3b and C3a and subsequent downstream formation of the membrane attack complex (MAC), which disrupts the cell membrane, destroying the microorganism. Each pathway also activates an inflammatory response, promoting chemotaxis and chemokinesis of leukocytes, activating the cells involved in immune response, and releasing inflammatory mediators from mast cells, facilitating the removal of the target particles [1,2].

The classical pathway is activated by antibodies, which form complexes with antigens on bacteria, viruses, or autoantigens. The C1 complex comprises three subunits: C1q, C1r, and C1s. C1q binds to the Fc portion of immunoglobulin G (IgG) or immunoglobulin M (IgM) in the immune complexes, resulting in the autoactivation of the serine proteases C1r, and C1s and triggers the cascade of reactions involving proteolytic cleavage of C4 and C2 leading to the formation of C3 convertase (C4bC2b) and cleavage of C3 (Figure 1). IgG1, IgG3, and IgG2 activate complements, while IgG4 is not involved in the classical pathway [1,2].

The lectin complement pathway recognizes the surface carbohydrate patterns on microorganisms. It is activated similarly to the classical pathway, except that the carbohydrate-binding proteins (lectins) replace the antibodies. Lectins (ficolins, collectins, mannose-binding lectin [MBL]) bind to mannose on bacterial surfaces, activating MBL-associated serine proteases (MASPs), cleaving C4 and C2, forming C3 convertase (C4bC2b) and MAC downstream [1,2].

In contrast, the alternative complement pathway does not require antibodies or lectins for activation. Instead, C3 is constantly self-activated to C3b at a low level, a process called C3 tick-over, amplifying in the presence of damaged host cells or microbes. In the absence of targets, it is inactivated quickly by plasma regulators and removed from circulation without harming healthy human cells. When the target particle is present, the activated C3 binds factor B (FB), which is cleaved by factor D (FD) to form C3 convertase (C3bBb). Properdin (P) then stabilizes the C3 convertase (C3bBbP), initiating an amplification loop, activating more C3 to C3b, and forming MAC [1,2].

Pro-inflammatory anaphylatoxins, such as C3a, C4a, and C5a, are generated and released as part of the cascade. The chemotactic properties of these fragments enable the recruitment of immune cells to the activation site and induce mast cell and basophil degranulation, releasing vasoactive and chemoattractant mediators [1,2].

The relatively short half-lives of the binding sites of C4b and C3b and the unstable nature of the complement enzymes C4b2b and C3bBb serve as passive control of the cascade, preventing repeated and prolonged activation. Multiple soluble and cell-bound complement inhibitors and inactivators mediate active control of the cascade, occurring at three main stages - activation or initiation, amplification (formation of convertases), and the formation of the terminal MAC (Figure 1). C1 inhibitor (C1Inh) controls the activation of classical and lectin pathways. It binds to the C1r and C1s components of the C1 complex, disassociating them from immunoglobulin-bound C1q, and blocks the active sites of MASPs. C4b-binding protein (C4BP) regulates the C4b-containing convertases in classical and lectin pathways, and factor H (FH) controls the C3b-containing convertases in the alternative pathway. FH also acts as a cofactor for factor I (FI), which cleaves and inactivates C3b and C4b. Complement receptor 1 (CR1; CD35) binds C1q, C3b, C4b, and MBL. Furthermore, cell-bound decay-accelerating factor (DAF; CD55) also contributes to the inactivation of convertases, and membrane cofactor protein (MCP; CD46) acts as a cofactor for FI. Protectin (CD59) and S protein inhibit the membrane-bound and fluid-phase MAC [1,2].

Role of complements in the pathophysiology of human diseases

Disorders involving the complement factors, or the regulatory proteins have been implicated in the pathogenesis of several diseases. Deficiencies or dysfunction of the complement factors lead to an impaired immune response, causing susceptibility to recurrent bacterial infections or autoimmune diseases, such as systemic lupus erythematosus (SLE). On the other hand, deficiencies or dysfunction of the regulatory proteins can cause an overheated immune response, causing exaggerated inflammation and tissue damage. Furthermore, autoantibodies against specific complement components leading to complement activation are also seen in SLE and other autoimmune disorders [3].

Primary (inherited) complement deficiencies are rare, often involving a single complement factor or a regulatory protein that disrupts the downstream cascade, causing under or overactivation. Deficiencies of the early complement factors of the classical pathway (C1q, C1r, C1s, C2, and C4) are usually associated with the development of autoimmune diseases. Accumulation of cellular debris and impaired clearance of immune complexes due to defective apoptosis are considered the underlying mechanisms for autoimmunity [3]. On the other hand, deficiencies of properdin or the complement factors of the terminal pathway (C5-C9) can cause recurrent bacterial infections [4].

Secondary (acquired) complement deficiencies are more common and most frequently result from autoantibodies' activation of the classical pathway and increased consumption of complement factors. Typical examples include SLE, antiphospholipid syndrome (APS), cryoglobulinemia, primary Sjogren's syndrome, and other immune complex-mediated systemic vasculitides. C3, C4, and total hemolytic complement (THC or CH50) are low in these disorders, signifying activation of the classical pathway [3].

Complement activation products have also been implicated in other inflammatory and autoimmune diseases. Immunofluorescence studies of histology samples in IgA nephropathy and Henoch-Schönlein purpura (HSP) nephritis primarily show MBL, MASP, and C4d deposits signifying involvement of lectin pathway in the pathogenesis [5].

In a study involving 120 patients with anti-neutrophil cytoplasmic antibody (ANCA)-associated vasculitis, plasma levels of C3a, C5a, soluble C5b-9, and Bb were significantly higher in the active stage of the disease along with lower levels of properdin pointing toward the involvement of the alternative pathway in the pathophysiology of ANCA-associated vasculitis [6].

Activation of the classical pathway is also thought to be involved in the pathogenesis of rheumatoid arthritis (RA). Although C3 and C4 levels are usually normal or high, the levels of complement factors in the synovial fluid are low, along with the high levels of complement cleavage products such as C3a, C5a, and C5b-9 [3,7].

C4b, C3b, and MAC are also seen in the muscles and skin biopsies of patients with dermatomyositis and other inflammatory myopathies [3]. Several chronic inflammatory neurological and ophthalmological diseases are associated with complement activation and consumption. Complement activation products and immune complex depositions have been traced in a variety of locations, including myelinated fibers of patients with Guillain-Barré syndrome and chronic inflammatory demyelinating polyneuropathies, nerve terminals of patients with myasthenia gravis, demyelinating lesions of patients with multiple sclerosis and neuromyelitis optica spectrum disorders, neuroglial cells of patients with amyotrophic lateral sclerosis and Huntington's disease, as well as cerebral amyloid plaques of patients with Alzheimer disease [8].

Diseases associated with classical pathway disorders

Table 1 summarizes the primary conditions associated with disorders of the classical pathway. CH50 assay is a reliable screening tool to detect the homozygous deficiencies of the integral complement factors of the classical pathway.

**Table 1:** Diseases associated with classical pathway disorders.

Complement disorders	Associated diseases
C1q deficiency	Systemic lupus erythematosus (SLE)
	Lupus-like illness
	Recurrent encapsulated bacterial infections
Anti-C1q autoantibody	SLE
	Lupus nephritis
	Hypocomplementemic urticarial vasculitis syndrome
	Mixed connective tissue disease
	Felty's syndrome
	Hepatitis C virus infection
Primary C1r and C1s deficiencies	SLE
	Lupus-like illness
	Lupus nephritis
C4 deficiency	Lupus-like illness
	SLE
	Juvenile idiopathic arthritis
	Immune complex glomerulonephritis
	Immunoglobulin A (IgA) nephropathy
	Henoch-Schönlein purpura (HSP)
	Noninfectious hepatitis
	Wegener's granulomatosis
C2 deficiency	SLE
	Recurrent infections with encapsulated bacteria
C3 deficiency	Severe encapsulated bacterial infections (*Streptococcus pneumoniae*, *Haemophilus influenzae*, and *Neisseria meningitidis*)
	Immune complex-mediated glomerulonephritis
C4 nephritic factor	SLE
	Membranoproliferative glomerulonephritis
	Poststreptococcal glomerulonephritis
C1 inhibitor deficiency (inherited and acquired)	Hereditary angioedema
	Acquired angioedema

Among the primary deficiencies of the components of the C1 complex, C1q deficiency is the most common. SLE or lupus-like illness is the most common autoimmune disease associated with homozygous C1q deficiency. Typically, these patients have high titers of antinuclear antibodies (ANA), particularly anti-Ro antibodies, with a low frequency of anti-double-stranded deoxyribonucleic acid (anti-dsDNA) antibodies. Complement C3 and C4 are normal. Photosensitivity, oral ulcers, arthralgia, glomerulonephritis, and neurological disease are common manifestations. Recurrent bacterial infections with encapsulated organisms have been reported in nearly 40% of the cases of primary C1q deficiency [9].

Anti-C1q autoantibody, triggered by environmental factors in genetically susceptible individuals, is strongly associated with SLE and lupus nephritis. Complement C3 and C4 levels are typically low, and anti-dsDNA antibodies are usually positive [10]. Nearly all patients with hypocomplementemic urticarial vasculitis syndrome (HUVS) have positive anti-C1q antibodies [11]. High titers of anti-C1q antibodies are also found in patients with mixed connective tissue disease, Felty's syndrome, and hepatitis C virus infection [12,13].

Primary C1r and C1s deficiencies are extremely rare and primarily present with recurrent bacterial, viral, and fungal infections, SLE, lupus-like illness, or lupus nephritis. Serum levels of C4, C2, C1-inhibitor, and C3 are typically high, while C1q is normal. Mortality is very high at a young age [14].

Complete C4 complement deficiency involving both C4 isoforms (C4A and C4B) is rare and often presents as early onset of lupus-like illness. Partial deficiency affecting one of the C4 isotypes is more common and is associated with SLE, juvenile idiopathic arthritis, and immune complex glomerulonephritis [15]. There are also reports suggesting an association of C4 deficiency with IgA nephropathy, chronic forms of noninfectious hepatitis, membranous nephropathy, subacute sclerosing panencephalitis, HSP, and Wegener's granulomatosis [3,16].

C2 bridges the classical and the lectin pathways to protect against microbes, especially encapsulated bacteria, and remove immune complexes. C2 deficiency is considered the most common complement deficiency, with milder manifestations, including a lower penetrance of SLE, compared to the other complement deficiencies. C2-deficient individuals are more likely to develop recurrent infections with encapsulated bacteria [17].

The complete deficiency of C3, which mediates the opsonization of microbes, typically results in severe encapsulated bacterial infections, especially *Streptococcus pneumoniae*, *Haemophilus influenzae*, and *Neisseria meningitidis*, and immune complex-mediated glomerulonephritis at a young age. SLE is very rare in isolated C3 deficiency. Patients with partial C3 deficiency usually have no significant clinical presentations [18].

Autoantibodies to the C4 component of the classical pathway's C3 convertase (C4b2b), the C4 nephritic factor (C4NeF), causes stabilization of C3 and C5 convertases and consumption of C3, leading to recurrent bacterial infections secondary to the low C3. It has also been associated with SLE, membranoproliferative glomerulonephritis, and poststreptococcal glomerulonephritis [19].

Hereditary deficiency of the regulator of C1, the C1 inhibitor, increases the activation of C2 and C4. C1 inhibitor also controls bradykinin production by inhibiting kallikrein and active factor XII. When the C1 inhibitor is deficient, excessive bradykinin accumulates. Resultant vasodilation and increased vascular permeability lead to the development of hereditary angioedema. Acquired antibodies to C1 inhibitors lead to acquired angioedema, with clinical presentation similar to hereditary angioedema. Anti-C1 inhibitor antibodies are also seen in SLE patients with features of angioedema [20].

Diseases associated with alternative pathway disorders

AH50 assay is a screen for alternative pathway component deficiency. Table 2 summarizes the conditions associated with alternative pathway disorders.

**Table 2:** Diseases associated with alternative pathway disorders.

Complement disorders	Associated diseases
Properdin deficiency, factor D deficiency, and/or factor B deficiency	Recurrent bacterial infections
	Meningitis (Neisseria infections)
	Pneumonia
	Otitis media
Inherited factor H deficiency, inherited factor I deficiency, and/or inherited CD46 deficiency	Atypical hemolytic uremic syndrome (aHUS)
	Age-related macular degeneration (AMD)
	C3 glomerulopathy
	Dense deposit disease (DDD)
	Membranoproliferative glomerulonephritis
C3 nephritic factor	DDD
	Membranoproliferative glomerulonephritis
	Partial lipodystrophy
	Frequent infections with encapsulated bacteria

Inherited alternative pathway deficiencies are rare and have only been documented in a few cases. Properdin, FD, and FB deficiencies have been associated with recurrent bacterial infections, especially meningitis (*Neisseria *infections), pneumonia, or otitis media [21].

Deficiencies of the regulatory proteins of the alternative pathway are more common and can cause various disorders. Inherited FH, FI, and MCP (CD46) deficiencies cause uninhibited activation of alternative complement pathways, leading to low C3, FB, and AH50 levels. C4 is typically normal. The deficiencies are associated with atypical hemolytic uremic syndrome (aHUS), age-related macular degeneration (AMD), C3 glomerulopathy, dense deposit disease (DDD), and membranoproliferative glomerulonephritis [22-24].

C3 nephritic factor (C3NeF), an autoantibody that stabilizes the alternative pathway's C3 convertase (C3bBb), causes excessive C3 activation and consumption. It is associated with DDD, membranoproliferative glomerulonephritis, partial lipodystrophy, and frequent infections with encapsulated bacteria [25].

Diseases associated with lectin pathway disorders

Inherited deficiencies of the lectin pathways components most commonly involve deficiencies of MBL and, rarely, MBL-associated protease 2 (MASP2) and ficolin 3 (Table 3).

**Table 3:** Diseases associated with lectin pathway disorders.

Complement disorders	Associated diseases
Mannose-binding lectin (MBL) deficiency	Systemic lupus erythematosus (SLE)
	Rheumatoid arthritis (RA)
MBL-associated protease 2 (MASP2) deficiency	Severe pneumococcal pneumonia
	Ulcerative colitis
	Erythema multiforme bullosum
Ficolin 3 deficiency	Recurrent pulmonary infections

MBL, as a component of the lectin pathway, is involved in removing apoptotic debris. Deficiencies in MBL result in increased susceptibility and severity of a multitude of illnesses and predispose patients to developing autoimmune diseases such as SLE and RA. MASP2 deficiency is also linked with severe pneumococcal pneumonia, ulcerative colitis, and erythema multiforme bullosum. Ficolin 3 deficiency may cause recurrent pulmonary infections with long-term severe sequelae [3,26].

Diseases associated with terminal pathway and membrane attack complex disorders

The terminal pathway is shared by all three complement activation pathways, which converge at the C3 stage (Figure 1). Any deficiency in the component of MAC, i.e., C5-C9, predominantly predisposes patients to disseminated infection by Neisseria species (especially the meningococcus). It is also associated with autoimmune diseases such as SLE, RA, pyoderma gangrenosum, and scleroderma in a small number of patients (Table 4). Both CH50 and AH50 are low in terminal pathway deficiencies as these complement factors are common in both assays [27,28].

Inherited mutations of the regulatory protein CD59 cause loss of CD59 on the red blood cell (RBC) surface and are linked with the development of paroxysmal nocturnal hemoglobinuria (PNH), causing intravascular hemolysis and thrombosis. Inherited CD59 deficiency has also been linked with relapsing immune-mediated peripheral neuropathy. CD55 blocks extravascular hemolysis, and its deficiency has been linked with complement-mediated extravascular hemolysis in PNH [29,30].

**Table 4:** Diseases associated with terminal pathway and membrane attack complex disorders.

Complement disorders	Associated diseases
C5-C9 deficiency (any complement component)	Recurrent bacterial infections
	Meningitis (Neisseria infections)
	Systemic lupus erythematosus (SLE)
	Rheumatoid arthritis (RA)
	Pyoderma gangrenosum
	Scleroderma
Mutations of CD59	Paroxysmal nocturnal hemoglobinuria (PNH) (Intravascular hemolysis and thrombosis)
Inherited CD59 deficiency	PNH Relapsing immune-mediated peripheral neuropathy
Inherited CD55 deficiency	PNH (Complement-mediated extravascular hemolysis)

Workup and investigations

Where a complement deficiency is suspected, initial investigations are usually aimed at assessing the overall function of the classical and alternative pathways and, if clinically indicated, lectin pathways. Subsequent diagnostic investigations to determine individual complement deficiencies can involve quantitative assays to assess complement factor concentrations, functional screening tests to assess for functional activity, and quantification of autoantibodies against complements [31].

Functional Assessments of the Complement System

Traditionally, hemolytic assays have been used for functional assessment of the classical and alternative pathways. CH50 is used as a screening test to measure classical pathway activity. Serial dilutions of a sample incubated with immunoglobulin-sensitized sheep red cells are made. The results are reported as the reciprocal of the serum dilution that caused the lysis of 50% of the sheep red cells. A similar technique measures alternate pathway activity (AH50). However, instead of sheep red cells, rabbit or guinea pig erythrocytes, which preferentially activate the alternate pathway, are used [31,32]. The classical pathway activity can also be assessed by measuring neoantigen formed during the generation of MAC by nephelometric, turbidometric, or enzyme-linked immunosorbent assay (ELISA)-based tests [31,33].

Low CH50 or AH50 indicates the defect in specific complements of classical or alternate pathways and helps plan further quantitative analysis [31,32]. A low CH50 with a normal AH50 indicates the deficiency of complement components of the classical pathway, suggesting quantitative and functional assessment of C1, C2, C4, and C1 inhibitors. On the other hand, a low AH50 and a normal CH50 point towards alternate pathway defects. The assessment of alternate pathway complement factors, FB, FD, FH, and FI, is indicated in this situation. Simultaneous low levels of CH50 and AH50 are seen with deficiencies in complement factors of the terminal pathway (common to both classical and alternative pathways) involving C3, C5, C6, C7, C8, and C9. Measuring MBL might be indicated when CH50 and AH50 are normal, but a complement disorder is strongly suspected, such as in patients with recurrent infections. Low MBL suggests a lectin pathway component deficiency [31].

Quantitative Assessment of Complement System

Quantitative measurement of most individual complement components can be evaluated using various immunochemical and immunoprecipitation assays such as nephelometry, radioimmunoassay (RIA), and ELISA. ELISAs are commonly preferred for quantification of complement activation products such as C4a, C4d, C3a, C3c, C3d, Ba, Bb, and C5a. Complement regulating factors such as FB, FH, FI, FD, and other products derived from the various complement pathways can be assessed utilizing monoclonal antibodies that recognize and target specific epitopes [34]. More recently, the simultaneous quantification of complement proteins (C1q, C2, C3, C4, C5, FD, FP, FH, FI) and cleavage fragments (C4a, C3a, C5a, Bb, Ba, sC5b9) using multiplex ELISA assays has also been reported [35].

Autoantibodies targeting complement proteins have also been shown to be involved in several complement-related diseases. Detection of autoantibodies against complements such as C1q and C1 inhibitors, which are present in diseases such as SLE, is usually performed via immunoprecipitation assays such as ELISAs with the corresponding antigens [36-38]. Aside from ELISAs, other methods have also been reported, such as a semiquantitative hemolytic assay for the detection of C3NeF in the alternative pathway and functional assays assessing the neutralizing effect of autoantibodies against FH and C1 inhibitors [36].

Deficiency of a single complement component usually suggests a hereditary or functional disorder, while abnormalities with multiple components point toward complement consumption seen in immune complex-mediated diseases [31].

An Overview of the Specific Therapies

The pivotal role complements play in the pathophysiology of a broad spectrum of diseases makes these proteins an attractive target for therapeutics. Interestingly, complement deficiencies tend to be associated with increased susceptibility to autoimmune diseases, most prominently SLE [37-39]. Studies have suggested that this can be attributed to the role complements play in the processing and downstream activation of immune complexes, such that inhibiting any of these steps could inhibit immune complex processing and further exacerbate the underlying autoimmune disease. Additionally, inhibition of the complement system would further increase susceptibility to infections, which is concerning in patients who are already at an increased risk of infections due to immunosuppressive therapy such as corticosteroids [37-39].

However, complement activation has also been shown to be a key mediator of tissue damage in autoimmune diseases, such as SLE, where products such as C4b, C3b, C5a, and C3a have been implicated [40]. As such, the primary aim of anti-complement therapeutics should be to target the inhibition of cellular injury and prevention of the production of pro-inflammatory peptides.

Two classes of anti-complement therapies have been approved for therapeutic use: C5 inhibitors and plasma C1 inhibitors. Eculizumab, a humanized monoclonal antibody against C5, inhibits the cleavage of C5 into C5a and C5b, thus blocking the further downstream formation of C5a anaphylatoxins and the MAC [24]. Eculizumab has been approved for treating PNH, aHUS, relapsing or refractory myasthenia gravis, and aquaporin antibody-positive (AQP)-4 neuromyelitis optica spectrum disorder [24,41,42]. Ravulizumab (ALXN1210), a derivative of eculizumab, which also targets C5, has a substantially longer terminal half-life relative to eculizumab, allowing for longer dosing intervals during the treatment of PNH [43]. A recombinant human C1 inhibitor is utilized to treat hereditary angioedema [44].

Danicopan, an oral inhibitor of FD, and iptacopan, an oral FB inhibitor, have also been identified as alternative treatments for PNH and decrease intravascular and extravascular hemolysis [45,46]. Avacopan is an oral complement 5a receptor (C5aR) antagonist effective in treating ANCA-associated vasculitis [47].

Complement Receptor 1 (CR1/CD35) inhibits the classical, lectin, and alternative complement pathways. Recombinant soluble CR1 therapy has also demonstrated effectiveness in inhibiting excessive activation of C3 convertase in a limited trial of patients with DDD and C3 glomerulonephritis [48].

Another therapeutic option is supplementing complement proteins in patients with complement deficiencies. Whole plasma preparations have been reported to be successful in patients with C2 deficiencies [49]. Fresh frozen plasma has also been effective in patients with C1q deficiencies. However, effectiveness drops rapidly within two weeks, thus requiring regular plasma infusions [50]. While studies have shown the development and testing of recombinant C2 concentrate in vitro, purified or recombinant complement proteins still need to be made available for treatment [51].

Currently, the development of complement inhibitors remains a difficult task. Different approaches will likely be needed for different diseases, owing to the differing underlying pathophysiology and the parts of the affected complement pathways. Challenges faced in developing anti-complement therapies include the nature of the complement proteins as acute phase reactants, which results in these proteins being present at high concentrations with rapid turnover rates, which may result in high, frequent doses of inhibitors required [52]. The balance between potential adverse effects, such as increased susceptibility to infections, owing to the physiological roles played by the complement systems in our innate immunity, is also a key consideration. Despite the potential of complements as a target for therapeutic intervention, further studies are required to establish their role in treating various diseases.

## Conclusions

Complements are essential in protecting from microorganisms and removing cellular debris. The activated complement system has strong pro-inflammatory effects and, if left unregulated, can harm the normal organs. The complement system is usually tightly regulated to prevent excessive activation, which is necessary to avoid damage to normal host cells. Various systemic and inflammatory diseases such as SLE and ANCA-associated vasculitis cause excessive stimulation of complement pathways, accumulating the pro-inflammatory activated complement products in the target tissues and contributing to the end-organ damage in these conditions. Moreover, inherited and acquired deficiencies of the complement factors and the regulatory proteins can result in several systemic diseases and recurrent infections. However, such diseases are rare in day-to-day clinical practice and, unless suspected, can be easily missed. Knowledge of the role of complements in the pathophysiology of human diseases and a high index of suspicion is required to make the correct diagnosis. In recent years, complements have increasingly been recognized as therapeutic targets in several diseases, and targeted anti-complement therapies such as eculizumab have transformed the outcome of various diseases such as aHUS and PNH.
